# Impaired Skin Barrier Due to Sebaceous Gland Atrophy in the Latent Stage of Radiation-Induced Skin Injury: Application of Non-Invasive Diagnostic Methods

**DOI:** 10.3390/ijms19010185

**Published:** 2018-01-08

**Authors:** Hyosun Jang, Hyunwook Myung, Janet Lee, Jae Kyung Myung, Won-Suk Jang, Sun-Joo Lee, Chang-Hwan Bae, Hyewon Kim, Sunhoo Park, Sehwan Shim

**Affiliations:** 1 Laboratory of Radiation Exposure & Therapeutics, National Radiation Emergency Medical Center, Korea Institute of Radiological and Medical Sciences, 75 Nowon-ro, Nowon-gu, Seoul 01812, Korea; hyosun327@gmail.com (H.J.); hwmyung@kirams.re.kr (H.M.); lys5017@kirams.re.kr (J.L.); tontos016@kirams.re.kr (J.K.M.); wsjang@kirams.re.kr (W.-S.J.); sjlee@kirams.re.kr (S.-J.L.); ssaybch@gmail.com (C.-H.B.); hw0227@kirams.re.kr (H.K.); 2 Department of Pathology, Korea Cancer Center Hospital, Korea Institute of Radiological and Medical Sciences, 75 Nowon-ro, Nowon-gu, Seoul 01812, Korea

**Keywords:** radiation, skin barrier, sebaceous gland, transepidermal water loss, skin pH

## Abstract

Radiation-induced skin injury can take the form of serious cutaneous damage and have specific characteristics. Asymptomatic periods are classified as the latent stage. The skin barrier plays a critical role in the modulation of skin permeability and hydration and protects the body against a harsh external environment. However, an analysis on skin barrier dysfunction against radiation exposure in the latent stage has not been conducted. Thus, we investigated whether the skin barrier is impaired by irradiation in the latent stage and aimed to identify the molecules involved in skin barrier dysfunction. We analyzed skin barrier function and its components in SKH1 mice that received 20 and 40 Gy local irradiation. Increased transepidermal water loss and skin pH were observed in the latent stage of the irradiated skin. Skin barrier components, such as structural proteins and lipid synthesis enzymes in keratinocyte, increased in the irradiated group. Interestingly, we noted sebaceous gland atrophy and increased serine protease and inflammatory cytokines in the irradiated skin during the latent period. This finding indicates that the main factor of skin barrier dysfunction in the latent stage of radiation-induced skin injury is sebaceous gland deficiency, which could be an intervention target for skin barrier impairment.

## 1. Introduction

Skin injury due to radiation therapy or after a nuclear or radiological event can take the form of serious cutaneous damage [1]. Skin injury by irradiation has been documented globally in all major radiation accidents over the last 15 years [2] and occurs in about 95% of patients receiving radiation therapy for cancer [3]. Skin injury negatively affects the quality of a patient’s life because of severe pain and premature interruption of radiation treatment, which in turn may impair control of the disease [4]. Moreover, skin injuries by radiation exposure have specific characteristics, including delayed onset of clinical problems post-irradiation. The asymptomatic periods in radiation-induced skin injury are classified as the latent stage. After the latent stage, the acute stage of radiation-induced skin injury begins, which is characterized by erythema, edema, pigment change, and depilation. The chronic stage includes delayed ulcers, fibrosis, and telangiectasia that present weeks to a year after radiation exposure [4,5]. No effective preventive measure or treatment for radiation-induced cutaneous damage is currently available, except for conservative managements [6]; thus, several patient deaths have been reported [7,8].

Early diagnosis of radiation-induced skin injury is vital because a rapid diagnosis determines the progress and/or success of treatment. Diagnostic methods for radiation-induced injuries are based on visible clinical symptoms, numerical dosimetry reconstruction, cytogenetic analysis, and other physical parameters. Diagnosis based on visible clinical symptoms is easy and convenient compared with other methods, but is limited by the clinically latent periods. Radiation dosimetry, which analyses the absorbed radiation dose and damage based on a mathematical model [9,10], has restrictions related to exposure type, distance of source, and exposure lesion; requires a considerable time or invasive biopsy sample; and obtains inaccurate data on local exposure. Therefore, a novel diagnostic method, especially for application in the latent stages, is warranted.

Furthermore, the skin provides a vital barrier against a harsh external environment and plays a critical role in the modulation of skin permeability and hydration. Skin barrier disruption is characterized by increased skin pH levels, transepidermal water loss (TEWL), and decreased epidermal hydration, and is associated with the development of inflammatory skin diseases, such as atopic dermatitis [11,12]. The skin barrier is affected by cornified envelope, skin surface lipid, and serine protease. The cornified envelope, which is mainly composed of filaggrin (FLG) and involucrin (IVL), surrounds corneocytes and provides the mechanical strength and rigidity of the skin, thereby protecting the host from injury [13,14]. The skin surface lipid is composed of both sebaceous gland (SG)- and keratinocyte-derived lipids. SG-derived lipids, which are synthesized by sebocytes, are secreted to the surface of the stratum corneum, and are essential in skin and hair coat waterproofing. Additionally, their antioxidative and antimicrobacterial properties have led to the assumption that they are also vital in maintaining the epidermal barrier [15,16,17]. Keratinocyte-derived lipids are synthesized mainly by fatty acid synthase (FASN) and 3-hydroxy-3-methyl-glutaryl-coenzyme A reductase (HMGCR) in keratinocytes, and play a role in the maintenance of the epidermal barrier [18,19]. The epidermis contains serine protease, such as kallikrein (KLK) 5 and KLK7 [20,21], which acts on the homeostasis of skin barrier function; the hyperactivity of serine protease degrades junctional protein [22] and induces inflammatory cytokines in keratinocyte [23]. Following barrier disruption, serine protease activity in the stratum corneum increases and serine protease inhibition accelerates the normalization of permeability barrier function [24]. However, information on the change in skin barrier, including the cornified envelope, skin surface lipid, and serine protease, in the latent stage of radiation-induced skin injury is limited.

In this study, we developed a non-invasive diagnostic method to evaluate skin barrier function, which could be applied during the asymptomatic stage of radiation-induced skin injury. We also investigated the underlying mechanisms of skin barrier dysfunction in the latent stage.

## 2. Results

### 2.1. Radiation Exposure Results in the Development of Skin Barrier Dysfunction in the Latent Stage 

**Figure 1:**
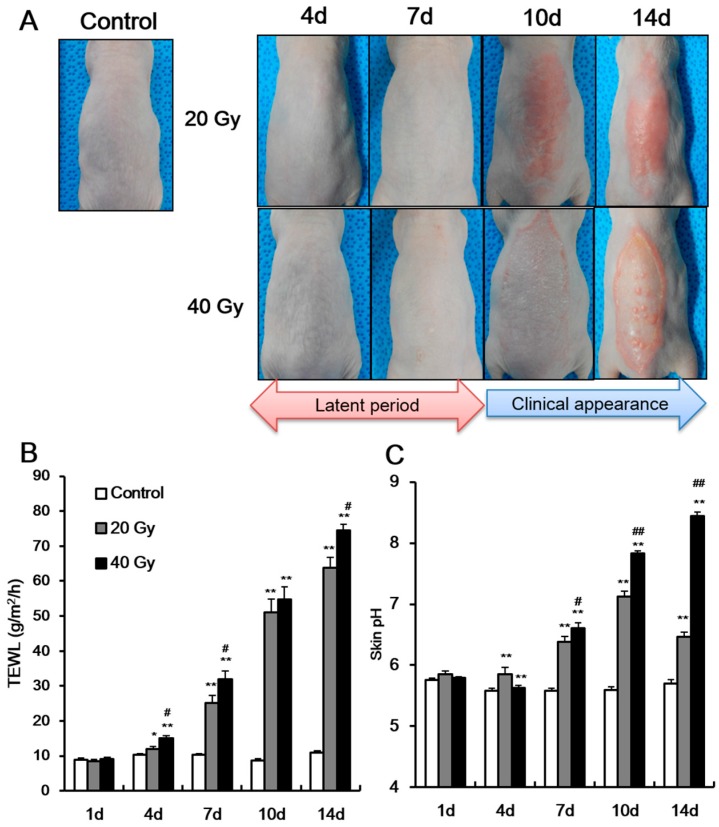
Increase of transepidermal water loss and skin pH in the latent stage of irradiated skin. (**A**) Phenotype and the levels of (**B**) transepidermal water loss (TEWL), and (**C**) skin surface pH of 0 (control), 20, and 40 Gy irradiated skin of SKH1 mice for 14 days (shown as “d” in figure body). Data are presented as the mean ± standard error of the mean. *n* = 5 mice for each group. * p < 0.05 and ** p < 0.01 compared with the control group; ^#^ *p* < 0.05 and ^##^ *p* < 0.01 compared with the 20 Gy group.

To detect dermatological phenotype alteration due to irradiation in a mouse model, we evaluated the skin of SKH1 mice that received a single dose of 20 or 40 Gy local irradiation for 14 days. The skin of 20 and 40 Gy groups showed no signs until 7 days after irradiation (Figure 1A). At 10 days, clinical symptoms appeared in both groups (Figure 1A). Thus, the latent stage in 20 and 40 Gy irradiated mouse model was 7 days.

To evaluate the skin barrier function in the latent stage, we measured the TEWL and skin pH in the two irradiated skin groups (i.e., 20 and 40 Gy) and in non-irradiated skin (control). Using non-invasive analysis methods, previous studies showed that TEWL and skin pH increase when the skin barrier is impaired, such as in dermatological diseases [11,12]. In our study, TEWL and skin pH in the 20 and 40 Gy irradiated groups significantly increased compared with those in the control group at 4 days (latent stage) (Figure 1B,C). In addition, a dose-dependent difference in TEWL levels at 4 days (Figure 1B) was observed. At 7 days after irradiation, the TEWL and skin pH in the irradiated skin were greater than those at 4 days (Figure 1B,C). A dose-dependent difference in TEWL and skin pH at 7 days was also noted (Figure 1B). These results indicate that skin barrier dysfunction appears in the latent stage, and impaired skin barrier could be detected in the latent stage using non-invasive methods.

After the latent stage, the clinical symptoms of the skin appeared differently between the groups. The 20 Gy irradiated group displayed an erythematous, edematous lesion (Figure 1A), whereas the 40 Gy irradiated group showed dry desquamation, erythema, moist desquamation, and papule lesion (Figure 1A). As clinical symptoms increased in severity, TEWL and skin pH also increased. Moreover, the skin pH in the 40 Gy irradiated group increased to >8 because some parts of the epidermal layer was removed by desquamation and scratching. The skin pH in the 20 Gy irradiated group did not exceed 8. The increased TEWL and skin pH in the latent stage implies the possibility of a new diagnostic method for radiation-induced skin injury during asymptomatic periods.

### 2.2. Skin Barrier Components Are Upregulated in Irradiated Skin in the Early Stage 

**Figure 2:**
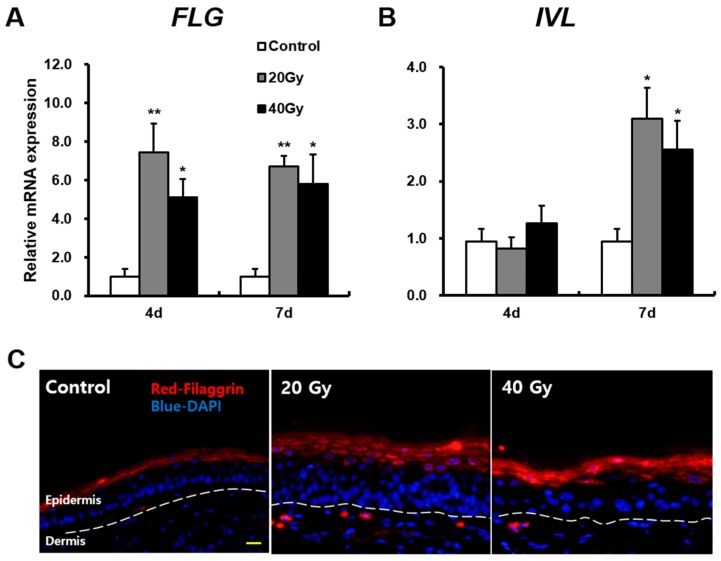
Increased fundamental proteins of the skin barrier in the latent stage on irradiated skin. mRNA levels of (**A**) *filaggrin (FLG)*, (**B**) *involucrin (IVL)* in the skin of 0 (Control), 20, and 40 Gy irradiated mice at 4 and 7 days. (**C**) Immunofluorescence stain of FLG in the irradiated skin of 20 and 40 Gy groups at 7 days. Scale bar = 20 μm. Data are presented as the mean ± standard error of the mean. *n* = 5 mice for each group. * p < 0.05 and ** p < 0.01 compared with the control group.

To explore the mechanisms of skin barrier defects in the latent stage, we first measured *FLG* and *IVL* expressions in the irradiated skin. FLG and IVL are part of the skin barrier and act on fundamental proteins in terminal epidermal differentiation [13,25]. The mRNA level of *FLG* in the 20 and 40 Gy irradiated skin groups significantly increased compared with that in the control group at 4 and 7 days after exposure (Figure 2A). Immunofluorescence staining showed that FLG existed in the stratum corneum in the control group and was expressed more extensively within the upper layer of the stratum granulosum in the 20 and 40 Gy irradiated skin groups (Figure 2C). Additionally, the mRNA level of *IVL* in the 20 and 40 Gy irradiated skin groups significantly increased compared with that in the control group at 7 days (Figure 2B). These results suggest that increased FLG and IVL expressions could be a comprehensive effect of radiological exposure related to skin barrier dysfunction.

### 2.3. Early Change in Sebaceous Gland (SG) Impairs the Skin Barrier of Irradiated Skin in the Latent Stage

**Figure 3:**
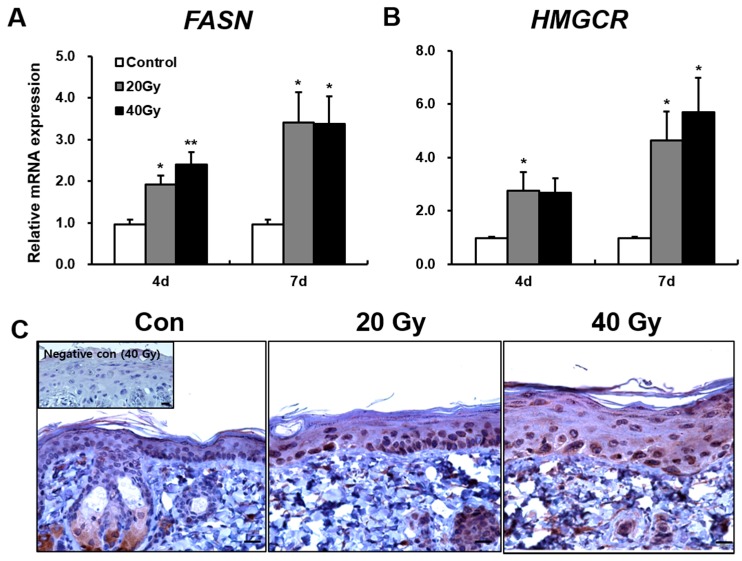
Increased keratinocyte-derived lipids synthesis enzymes in the latent stage of the irradiated skin. mRNA levels of (**A**) *Fatty acid synthase (FASN)* and (**B**) *3-hydroxy-3-methylglutaryl-CoA reductase (HMGCR)* in the irradiated skin of 0 (Control), 20, and 40 Gy groups at 4 and 7 days after exposure; (**C**) Immunohistochemistry of FASN in the skin of 0 (Control; Con), 20, and 40 Gy irradiated mice at 7 days. Insert: negative control. Scale bar = 20 μm. Data are presented as the mean ± standard error of the mean. *n* = 5 mice for each group. * p < 0.05 and ** p < 0.01 compared with the control group.

As protein components of the skin barrier were not impaired in the irradiated skin, we investigated other causes of skin barrier disruption. Skin lipid barrier is a primary factor of skin barrier homeostasis. To investigate skin lipid alteration by irradiation, we analyzed the synthesis enzymes of keratinocyte-derived lipids in the irradiated skin. FASN and HMGCR are found in keratinocyte and are involved in the synthesis of fatty acid and cholesterol, which are components of keratinocyte-derived lipids. The mRNA levels of *FASN* in the 20 and 40 Gy irradiated skin groups significantly increased compared with those in the control group at 4 and 7 days after exposure. No difference in *FASN* levels between the 20 and 40 Gy groups was observed (Figure 3A). At 7 days, FASN was strongly expressed in the lower stratum spinosum and stratum basale layers in the 20 and 40 Gy irradiated skin groups (Figure 3C). Moreover, the mRNA levels of *HMGCR* in the 20 and 40 Gy irradiated skin groups significantly increased compared with those in the control group at 7 days (Figure 3B). No difference in *HMGCR* expression between the 20 and 40 Gy groups was noted (Figure 3B). These results suggest that increased FASN and HMGCR expressions in irradiated skin could be a comprehensive effect for skin barrier recovery.

**Figure 4:**
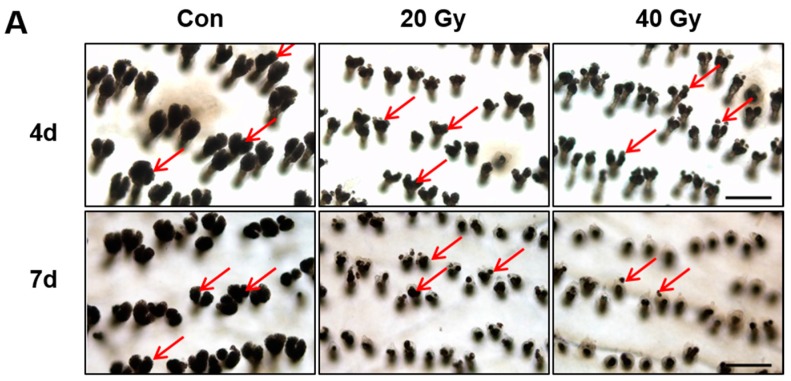

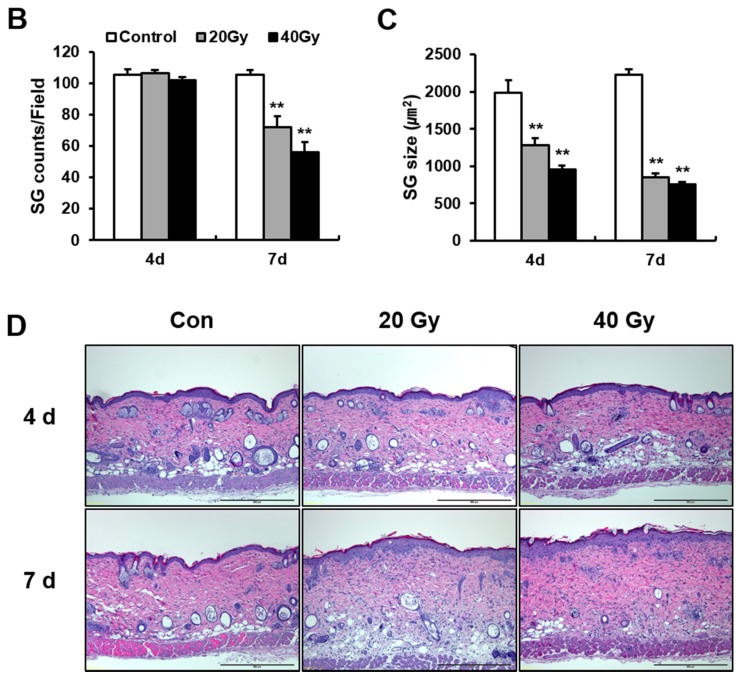
Decrease of sebaceous gland size and number by irradiation in the latent stage. (**A**) Optical microscopy view of sebaceous glands (SGs) in the epidermal sheet of 0 (Control; Con), 20, and 40 Gy irradiated skin at 4 and 7 days. Scale bar = 200 μm. Analysis of (**B**) the number of SGs and (**C**) the size of SGs in the irradiated skin of 0 (Control), 20, and 40 Gy groups at 4 and 7 days. (**D**) H&E stain of the 0 (Control; Con), 20, and 40 Gy irradiated skin at 4 and 7 days. Scale bar = 500 μm. Data are presented as the mean ± standard error of the mean. *n* = 5 mice for each group. ** p < 0.01 compared with the control.

To investigate SG-derived lipid alteration, we performed an analysis of the SG phenotype in irradiated skin using the epidermal sheet and SG visualization using optical microscopy (Figure 4A). The size of the SG in the 20 and 40 Gy irradiated skin groups significantly decreased compared with that in the control group at 4 and 7 days (Figure 4C). In addition, the number of SGs in the skin markedly decreased in the 20 and 40 Gy irradiation groups at 7 days (Figure 4B). These results suggest that SG atrophy in the latent stage impairs the skin barrier in the irradiated skin model. The atrophy of SGs and hair follicles in irradiated skin at 4 and 7 days could be detected in histological analysis (Figure 4D). Hyperkeratosis and subcutaneous edema appeared in the 20 and 40 Gy irradiated skin groups at 4 and 7 days (Figure 4D). Based on the results, histological changes in the irradiated skin had already progressed in the early stage. 

### 2.4. Serine Protease Hyperactivity Exacerbates Skin Inflammation in Irradiated Skin

**Figure 5:**
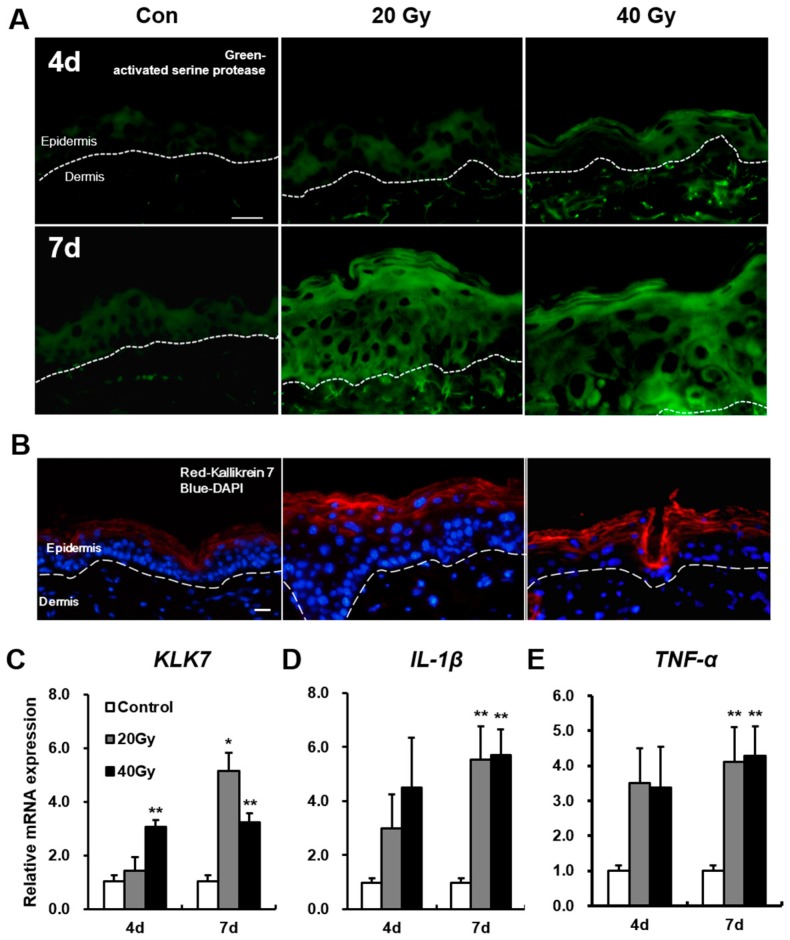
Upregulation of serine protease (KLK7) activity and expression in the latent stage of the irradiated skin. (**A**) Assay of serine protease activity by in situ zymography in the skin of 0 (control; Con), 20, and 40 Gy irradiation at 4 and 7 days. Scale bar = 20 μm; (**B**) Immunofluorescence stain of KLK 7 in the irradiated skin of 0 (control; Con), 20, and 40 Gy at 7 days. Bar = 20 μm. mRNA levels of (**C**) *KLK7*; (**D**) *IL-1β*, and (**E**) *TNF-α* in the irradiated skin of 0 (control), 20, and 40 Gy at 4 and 7 days. Data are presented as the mean ± standard error of the mean. *n* = 5 mice for each group. * p < 0.05 and ** p < 0.01 compared with the control group.

To identify changes in serine protease by radiation exposure, we performed an assay of serine protease activity in the irradiated skin using in situ zymography. Serine protease activity in the 20 and 40 Gy irradiated skin groups progressively increased compared with that in the control group at 4 and 7 days (Figure 5A). KLK7, one of the serine proteases that exist in the epidermis, was noted in the stratum corneum of the non-irradiated skin and was widely extended to the stratum granulosum in the 20 and 40 Gy irradiated skin groups at 7 days (Figure 5B). The mRNA levels of *KLK7* in the 20 and 40 Gy irradiated skin groups significantly increased compared with those in the control group at 7 days (Figure 5C).

Subsequently, we investigated the level of inflammatory cytokines in the irradiated skin. The mRNA levels of *interleukin (IL)-1β* and *tumor necrosis factor (TNF)-α*, which are the main inflammatory cytokines induced by irradiation [26,27], in the 20 and 40 Gy groups significantly increased compared with those in the control group at 7 days (Figure 5D,E). These results indicate that serine proteases and inflammatory cytokines are upregulated in the latent stage of the irradiated skin. 

## 3. Discussion

In this study, we defined the latent stage in 20 and 40 Gy irradiation in a mouse model as 7 days after radiation exposure. No difference in the duration of the latent stage between the 20 and 40 Gy irradiated groups was noted. TEWL and skin pH, which are parameters of skin barrier function in dermatological disorders, increased in the latent stage. New and non-invasive diagnostic methods that analyze skin barrier function in radiation exposure have clinical benefits, such as real-time measurement and patient satisfaction. Environmental control limitation and the restrictions related to a single measurement should be considered in developing new diagnostic methods.

The skin subjected to a single dose of 40 Gy irradiation showed clinical symptoms, such as dry and moist desquamation and skin pH level >8 at 14 days after exposure. Erythema and an edematous lesion were observed in the 20 Gy irradiated skin with a skin pH of 7. Thus, the progress of clinical symptoms depends on the irradiation dose. Hence, therapeutic strategy should consider the irradiation dose.

We investigated the change in skin permeability and skin barrier components in irradiated skin in the latent stage. FLG and IVL significantly increased in the irradiated skin for the latent stage. Liao et al. reported that the expression of keratins, which provide a structural stability of corneocytes by contacting with FLG, changes in the early stage of the radio-dermatitis model [28]. FLG is particularly important in skin barrier formation because of its fundamental role in terminal epidermal differentiation and its implication in some of the most common dermatological diseases, such as atopic dermatitis and ichthyosis vulgaris [25]. An FLG-deficient mouse model showed spontaneous dermatitis with impaired skin barrier and delayed skin barrier recovery from irritation [25,29]. Moreover, external stimuli increased FLG and IVL expressions in the healthy skin of a human and mouse model; the authors indicated that such an outcome is a comprehensive effect of the skin normalizing the skin barrier [30,31].

Our data showed that FASN and HMGCR, which are lipid synthesis enzymes in keratinocytes, are upregulated in irradiated skin in the latent stage. These results suggest that keratinocyte-derived lipids also increase in the irradiated skin of mice. Experimental skin permeability disruption upregulates epidermal HMGCR expression and activity as well as cholesterol content, and subsequently recovers skin barrier functions [19]. The use of an HMGCR inhibitor in an impaired skin barrier delays recovery rate [32]. FASN is also upregulated by barrier disruption and is required for barrier permeability repair [18]. Therefore, we speculate that increased enzymatic activity of FASN and HMGCR could be a comprehensive effect that improves skin permeability in irradiated skin.

Subsequently, we performed epidermal separation in the irradiated skin to identify SG alteration due to radiation exposure. The size and number of SGs significantly decreased in the irradiated skin in the latent stage. Similarly, Jang et al. reported that SGs in skin with 40 Gy radiation exposure become rounder and smaller at 4 days compared to those of non-radiated skin based on a two-photon image [33]. In addition, we identified that atrophy of SG was noted in both 20 and 40 Gy single irradiations in the latent stage. SG-derived lipid, which generates pheromones and body odors [34], acts as a delivery system for antimicrobial lipids [35] and antioxidants [36]. Another possible function of the SG is skin permeability maintenance. SG depletion affects skin hydration [37], negatively influences the skin’s ability to repulse water, and increases susceptibility to UVB-induced apoptosis in the epidermis of a mouse model [38]. In addition, a decrease of SGs in patients with atopic dermatitis is frequently associated with skin barrier dysfunction [39]. Hence, skin barrier defects are related to SG dysfunction in the acute latent stage in irradiated skin.

Serine protease activity is regulated by pH and involves the maintenance of skin permeability by degrading epidermal junctional molecules, such as corneodesmosome and desmoglein 1 [22]. Serine protease hyperactivity by skin surface alkalization induces skin barrier dysfunction and results in skin inflammation [31,40]. A mouse model of overactivated serine protease shows skin barrier dysfunction and dermatological inflammation, including increased IL-1β and TNF-α expression in the skin [40]. IL-1β and TNF-α, which are potent pro-inflammatory cytokines, are increased by radiation exposure and mediate inflammatory reactions in the skin [26,27]. In our study, we identified increased skin pH and serine protease hyperactivity, including KLK7, in the irradiated skin in the latent stage. We also found that IL-1β and TNF-α increase in the latent stage.

In conclusion, our results demonstrate SG atrophy and impaired skin barrier with increased TEWL and skin pH in the latent stage in irradiated skin. These findings present a potential target for therapy in the treatment of radiation-induced skin injury. Moreover, skin barrier function assessment in the latent stage could be a novel diagnostic method in radiation exposure.

## 4. Materials and Methods

### 4.1. Mice

Specific pathogen-free (SPF) male SKH1-Hr/Hr hairless mice (7 weeks old) were obtained from Jackson Laboratories (Bar Harbor, ME, USA) and maintained under SPF conditions at the animal facility of Korea Institute of Radiological and Medical Sciences (KIRAMS). All mice were housed in a temperature-controlled room with a 12-h light/dark cycle, and food and water were provided ad libitum. The mice were acclimated for 1 week before experiments and assigned to the following groups: control (*n* = 15), 20 Gy (*n* = 15), and 40 Gy (*n* = 15). All animal experiments were performed in accordance with the institutional guidelines and were approved by the Institutional Animal Care and Use Committee of KIRAMS (IACUC permit number: KIRAMS2016-0010; approval date: 4 February 2016). 

### 4.2. Irradiation of the Skin

Animals were anesthetized with an intraperitoneal administration of 85 mg/kg alfaxalone (Alfaxan^®^; Careside, Gyeonggi-do, Korea) and 10 mg/kg xylazine (Rompun^®^; Bayer Korea, Seoul, Korea). The mice were irradiated with different doses using X-RAD 320 X-ray irradiator (Softex, Gyeonggi-do, Korea). For the local irradiation of the back skin, the mice were placed in a lateral recumbent position; the pulled skin was fixed by needles. Because the single irradiation of a high dose has been applied the model of radiation-induced skin injury in a mouse and minipig [33,41,42], the mice were irradiated with a single exposure to X-ray of 20 and 40 Gy at a dose rate of 2 Gy/min.

### 4.3. Analysis of Transepidermal Water Loss (TEWL) and Skin Surface pH

TEWL and skin pH were measured with a multiprobe adapter (CK Electronic, Cologne, Germany) at 24 ± 2 °C and 45 ± 5% relative humidity. 

### 4.4. RNA Extraction, Reverse Transcriptase Polymerase Chain Reaction (RT–PCR), and Real-Time PCR Quantification

Mouse skin tissues were immediately snap-frozen and stored at −80 °C until RNA extraction. Total RNA was isolated from the skin tissues using TRIzol reagent (Invitrogen, Carlsbad, CA, USA). cDNA was synthesized using AccuPower RT premix (Bioneer, Daejeon, Korea), according to the manufacturer’s protocol. Real-time RT–PCR was performed using a LightCycler 480 system (Roche, San Francisco, CA, USA). The primer sequences are provided as follows: *FLG*, F: 5′-GGACAACTACAGGCAGTCTTGAAGA-3′, R: 5′-CATTTGCATGAAGACTTCAGCG-3′, *IVL*, F: 5′-AATTGGAGAACCGGACACAG-3′, R: 5′-TCTTTCCACAACCCACAGG-3′, *FASN*, F: 5′-AGGGGTCGACCTGGTCCTCA-3′, R: 5′-GCCATGCCCAGAGGGTGGTT-3′, *HMGCR*, F: 5′-CTTGTGGAATGCCTTGTGATTG-3′, R: 5′-AGCCGAAGCAGCACATGAT-3′, *KLK7*, F: 5′-CTCCACAAAGACCCACGTCA-3′, R: 5′-GTTTTCCCCAGCAGGTCCTT-3′, *IL-1β*, F: 5′-GCAACTGTTCCTGAACTCA-3′, R: 5′-CTCGGAGCCTGTAGTGCAG-3′, *TNF-α*, F: 5′-GCCTCTTCTCATTCCTGCTT-3′, R: 5′-CACTTGGTGGTTTGCTACGA-3′, and *β-actin*, F: 5′-TCCCTGGAGAAGAGCTATGA-3′, R: 5′-CGATAAAGGAAGGCTGGAA-3′. The expression levels of each target gene, which were determined using the LightCycler 480 system software (Roche), were normalized to that of *β-actin*. Cycle threshold values were used to calculate the relative mRNA expression using the 2^−ΔΔCt^ method.

### 4.5. Histological Analysis (Immunohistochemistry and Immunofluorescence)

Skin samples were fixed in 10% formalin and embedded in paraffin or directly frozen at −80°C in Tissue-Tek O.C.T.™ (Sakura, Japan). Hematoxylin and eosin (H&E) was used to stain 4-μm sagittal sections for histological examination. For antigen retrieval, paraffin-embedded cross-sections were boiled in 1 mM of citrate buffer, pH 6, for 20 min for FASN immunodetection. The slide was treated with 0.3% hydrogen peroxide in methyl alcohol for 20 min to block endogenous peroxidase activity. After three washes in phosphate-buffered saline (PBS), the sections were blocked with 10% normal goat serum (Vector ABC Elite kit; Vector Laboratories, Burlingame, CA, USA) and allowed to react with an FSAN antibody (Santa Cruz, Dallas, TX, USA). After three washes in PBS, the sections were incubated with a horseradish peroxidase-conjugated secondary antibody (Dako, Carpinteria, CA, USA) for 60 min. Peroxidase reaction was developed using a diaminobenzidine substrate (Dako), which was prepared according to the manufacturer’s instructions, and the slides were counterstained with hematoxylin.

For immunofluorescence, cryosections of the skin sample were fixed with acetone for 10 min and blocked with 10% normal goat serum (Vector ABC Elite kit; Vector Laboratories, Burlingame, CA, USA). The slides were incubated overnight with mouse FLG antibody (Novus Biologicals, Littleton, CO, USA) and mouse KLK7 antibody (R&D systems, Minneapolis, MN, USA) at 4 °C. A signal was detected with a fluorescein isothiocyanate-conjugated secondary antibody (Dako), and nuclei were stained with 4′,6-diamidino-2-phenylindole (DAPI) (Vector Laboratories). The fluorescence signal was visualized with a microscope.

### 4.6. Epidermal Separation and Sebaceous Gland Analysis

To identify SG alteration by irradiation, we performed epidermal separation in the irradiated skin with a slight modification [43]. Briefly, epidermal sheets were peeled from the mouse’s back skin incubated with 5 mM ethylenediaminetetraacetic acid in PBS for 8 h. The SG of the epidermal sheet was visualized with a light microscope.

### 4.7. Serine Protease Activity Assessment by In Situ zymography

Frozen sections (5 μm) were rinsed with a washing solution (1% Tween 20 in distilled water) and incubated at 37 °C for 2 h with BODIPY-FI-casein (Invitrogen, Carlsbad, CA, USA) in distilled water (0.1 g/μL). Sections were subsequently rinsed with the washing solution, counterstained with DAPI, and examined at an excitation wavelength of 485 nm and an emission wavelength of 530 nm [22].

### 4.8. Statistical Analysis

All quantitative data are expressed as mean ± standard error of the mean. Statistical significances of the differences were evaluated by one-way analysis of variance (ANOVA) with Tukey’s multiple comparison test. A *p*-value of <0.05 was considered statistically significant.

## Abbreviations

TEWL	Transepidermal water loss
FLG	Filaggrin
IVL	Involucrin
SG	Sebaceous gland
FASN	Fatty acid synthase
HMGCR	3-Hydroxy-3-methyl-glutaryl-coenzyme A reductase
KLK	Kallikrein
TNF-α	Tumor necrosis factor-α
IL-1β	Interleukin-1β
RT–PCR	Reverse transcriptase polymerase chain reaction
PBS	Phosphate-buffered solution
